# Gas Sensing of Laser-Produced Hybrid TiO_2_-ZnO Nanomaterials under Room-Temperature Conditions

**DOI:** 10.3390/nano13040670

**Published:** 2023-02-09

**Authors:** Neli Mintcheva, Dinesh Kumar Subbiah, Marat E. Turabayev, Stanislav O. Gurbatov, John Bosco Balaguru Rayappan, Aleksandr A. Kuchmizhak, Sergei A. Kulinich

**Affiliations:** 1 Research Institute of Science and Technology, Tokai University, Hiratsuka 259-1292, Kanagawa, Japan; 2 Department of Chemistry, University of Mining and Geology, 1700 Sofia, Bulgaria; 3 Centre for Nanotechnology & Advanced Biomaterials (CeNTAB), School of Electrical & Electronics Engineering, SASTRA Deemed University, Thanjavur 613 401, Tamil Nadu, India; 4 Department of Mechanical Engineering, Tokai University, Hiratsuka 259-1292, Kanagawa, Japan; 5 Far Eastern Federal University, 690041 Vladivostok, Russia; 6 Institute of Automation and Control Processes, Far Eastern Branch of the Russian Academy of Science, 690091 Vladivostok, Russia

**Keywords:** laser ablation in liquid, hybrids TiO_2_-ZnO, gas sensing, ethanol, 2-propanol

## Abstract

The preparation method can considerably affect the structural, morphological, and gas-sensing properties of mixed-oxide materials which often demonstrate superior photocatalytic and sensing performance in comparison with single-metal oxides. In this work, hybrids of semiconductor nanomaterials based on TiO_2_ and ZnO were prepared by laser ablation of Zn and Ti plates in water and then tested as chemiresistive gas sensors towards volatile organics (2-propanol, acetaldehyde, ethanol, methanol) and ammonia. An infrared millisecond pulsed laser with energy 2.0 J/pulse and a repetition rate of 5 Hz was applied to Zn and Ti metal targets in different ablation sequences to produce two nano-hybrids (TiO_2_/ZnO and ZnO/TiO_2_). The surface chemistry, morphology, crystallinity, and phase composition of the prepared hybrids were found to tune their gas-sensing properties. Among all tested gases, sample TiO_2_/ZnO showed selectivity to ethanol, while sample ZnO/TiO_2_ sensed 2-propanol at room temperature, both with a detection limit of ~50 ppm. The response and recovery times were found to be 24 and 607 s for the TiO_2_/ZnO sensor, and 54 and 50 s for its ZnO/TiO_2_ counterpart, respectively, towards 100 ppm of the target gas at room temperature.

## 1. Introduction

Pulsed laser ablation in liquid (PLAL) is a simple, easy-to-use, and convenient method to generate diverse nanomaterials at a laboratory scale [1,2,3,4,5,6,7,8,9,10,11,12,13]. It is an environmentally friendly approach that has become increasingly popular over the last 2–3 decades for its ability to produce metallic, metal oxide, sulfide, and carbide nanoparticles (NPs), among others, with “clean” surface and controlled sizes, chemical composition, and morphology [1,2,3,4,5,6,7,8,9,10,11,12,13]. Typically, a laser beam is focused on a solid target that is immersed in a liquid medium, resulting in plasma, vapor, or molten metal drops ejected into the liquid phase. After further quenching and/or reaction with the liquid, such species form nuclei and clusters that then grow as NPs whose morphology, size, and chemical composition depend on the laser pulses, target, and liquid used [1,3,4,5,6,7,9,10,11,12,13,14,15]. When noble metals are ablated, pure metal NPs are generated [4,6], while the ablation of more reactive metals in water or organic solvents leads to the production of metal oxide NPs [4,6,7,10,11,12,13,14,15]. Because of extremely high-temperature gradient and quenching rates created in the ablation zone during PLAL, nanomaterials rich in defects, with metastable phases or unique morphologies are often formed [3,4,5,6,7]. This explains why such nanomaterials are potentially attractive for catalysis, photocatalysis, optics, and optoelectronics, as well as for gas sensing applications [3,4,5,6,7,8,9].

As a method, PLAL permits generating NPs of various metal oxides, including TiO_2_ and ZnO, the latter two being widely used and investigated as semiconductor nanomaterials for photocatalysis, photovoltaics, as well as for hydrogen generation and gas sensing applications [1,2,3,4,6,7,8,9,11,13,15,16,17,18,19,20,21,22,23,24]. Even though laser-processed TiO_2_ nanomaterials were often observed to be amorphous and with hydroxyl-rich surface, they demonstrated defective nature, typically small particle size, which was associated with photocatalytic and sensing behavior [2,8,13,17,18,19]. If necessary, the produced TiO_x_ NPs could be further laser-modified to change their size, structure, surface chemistry, and other properties [13,17,18,19,20]. Depending on the lasers used, their parameters, and liquid media, TiO_2_ NPs with different phases were reported. For instance, the ablation of metallic Ti plate by ns-pulsed laser with a wavelength of 1064 nm in water and methanol was reported to generate anatase [21,22]. When the second harmonic (532 nm) of the ns-pulsed laser was employed in water, it generated a mixture of rutile and anatase [23]. Interestingly, in presence of surfactants, TiO_2_ NPs with rutile phase were prepared [24,25]. Boutinguiza and coworkers demonstrated controlled preparation of phases (rutile or brookite) in TiO_2_ NPs, which was achieved through the use of different liquid media (water and ethanol, respectively) [26,27].

Irrespective of the lasers used, PLAL-generated ZnO NPs typically have a wurtzite structure, while their morphology, size, and surface defects were shown to be dependent on experimental conditions. Typically, ablation of Zn in neat water was reported to give rise to spherical ZnO NPs with different sizes [10,14,15,28,29,30,31]. To reduce the size of ZnO NPs and stabilize them in water, various surfactants were proposed [32,33,34]. At the same time, ZnO nanorods with different aspect ratios were obtained by ablating metal Zn plates in water with millisecond-long pulses [35]. Predictably, ZnO spheres and nanorods generated by ns-pulsed and ms-pulsed lasers demonstrated different properties as photocatalysts and gas sensors [31,35,36,37].

As can be expected, enhanced gas sensing was reported for hybrids of both laser-prepared ZnO and TiO_2_ NPs with other semiconductor oxides [38,39,40,41]. Nevertheless, apart from our previous report [15], little work was devoted to ZnO-TiO_2_ hybrids produced by PLAL, even though the technique allows for the convenient preparation of hybrid metal-oxide nanomaterials [15,42]. Previously, we demonstrated that PLAL-generated TiO_x_/ZnO nanomaterials have properties dependent on laser parameters used [15], which makes us believe that such ZnO-TiO_2_ nanomaterials generated under different conditions may demonstrate sensitivity and selectivity towards different gases.

Therefore, in the present work, we prepared two composites by ablating Ti and Zn plates in water, TiO_2_/ZnO and ZnO/TiO_2_, and tested them as chemiresistive gas sensors at room temperature. This is the first work on gas-sensing of PLAL-prepared ZnO-TiO_2_ hybrids. It is shown that the experimental conditions used to prepare such nanomaterials influence not only their morphology and composition but also their gas-sensing performance.

## 2. Experimental Procedure

### 2.1. Preparation of Hybrid ZnO-TiO_2_ Nanomaterials

**Figure 1:**
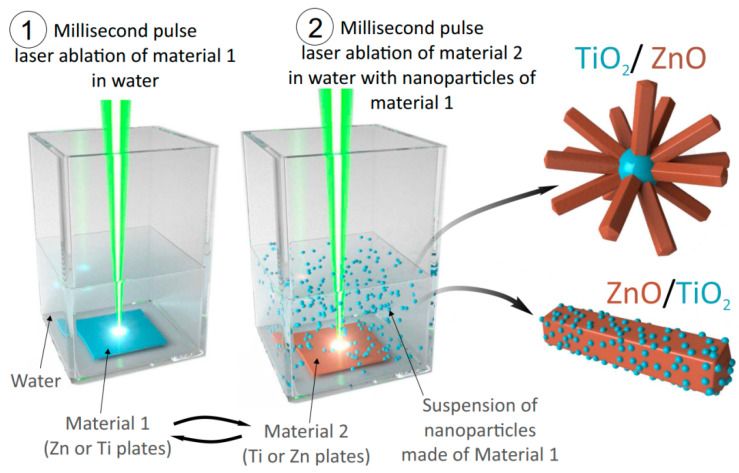
Schematic illustration of preparation procedure of hybrid nanomaterials ZnO/TiO_2_ and TiO_2_/ZnO using PLAL.

The experimental setup used to prepare materials is schematically shown in Figure 1. A millisecond pulsed Nd:YAG laser with a wavelength of 1064 nm, pulse peak power of 1 kW, pulse width of 2 ms, and repetition rate of 5 Hz was applied to ablate metal targets placed in a quartz cuvette. The beam was focused on the target surface by a lens with a focal length of 9.0 cm, with the diameter of ablated area being ~150 μm. More detailed descriptions of the setup and procedures used can be found elsewhere [35]. A zinc plate (99.5% purity, 2 mm in thickness) was fixed in the cuvette filled with 15 mL of deionized water, after which the target was ablated for 30 min. Then, the Zn plate was replaced with a Ti plate (99.5% purity, 0.5 mm thick), and irradiation was continued for another 30 min. The prepared sample was denoted as ZnO/TiO_2_ (Figure 1). The second sample, TiO_2_/ZnO, was prepared by first ablating Ti plate in 15 mL of deionized water for 30 min, followed by ablation of Zn plate in the TiO_2_ dispersion for another 30 min (Figure 1). During the preparation, the temperature of dispersions was found to rise up to ~67 °C, which is explained by heating caused by millisecond-long pulses. The as-prepared samples were centrifuged, the supernatant was removed, and NPs were concentrated in a volume of 1 mL. Then, the colloids were drop-cast on copper grids, on Si wafers, and on interdigitated electrodes for transmission electron microscopy (TEM), X-ray diffraction (XRD), X-ray photoelectron spectroscopy (XPS), and gas sensing test, respectively.

### 2.2. Characterization of Hybrids

TEM observations were carried out in a Hitachi HF-2200 microscope. Both samples were examined by X-ray photoelectron spectroscopy (XPS, Quantum 2000, ULVAC-PHI, Chigasaki, Japan) and X-ray powder diffraction (XRD, D8 Discover from Bruker, Leipzig, Germany), for which nanomaterials were dropcast on Si wafers.

### 2.3. Sensing Measurements

All the VOCs used in gas sensing tests, namely 2-propanol, acetaldehyde, ethanol, methanol, and ammonia solution, were purchased from Merck, India. To prepare sensors, the laser-prepared nanomaterials were dropcast on commercially available interdigitated electrodes (G-IDEAU5) supplied by Metrohm-DropSens (Oviedo, Spain). Gas sensing characteristics of the prepared samples were studied using a custom-made gas testing chamber integrated with high resistance electrometer (Keithley 6517B, Tektronix, Beaverton, OR, USA) [43,44]. The electrical contacts were established on interdigitated electrodes using gold wire bonder with a wire thickness of 20 μm [44]. The sensing elements were then placed inside the gas sensing chamber and connected to the high-resistance electrometer with a bias voltage of 5 V. The gas testing chamber was filled with ambient air, and resistance was measured to establish the baseline resistance (*R*_a_) for the sensing studies.

The concentration of target gases was determined following the protocol and formulas previously published elsewhere [43,44]. A chromatographic syringe was used to inject the desired volume of targeted volatile organic compounds (VOCs) into the test chamber. Subsequently, the sensing element’s resistance was monitored continuously until it reached a steady state value in the presence of desired concentration of VOC. The static liquid–gas distribution was adapted to calculate the concentration of VOCs from the injected target analytes, as given in Equation (1) [43]:

(1) $$Q=\frac{V\varphi M}{22.4dp}\times 10^{-6}\times \frac{273+T_{R}}{273+T_{B}}$$

where *Q* is the liquid volume of VOC (L), *V* is the volume of the gas testing chamber (L), *φ* is the required gas volume fraction, *d* is the specific gravity of the VOC solution (g/cm^3^), *p* is the purity of the specific VOC solutions, *M* is the molecular weight of the target molecule (g/mol), and *T_R_* and *T_B_* are the temperatures of ambient and testing chambers environment (°C).

Once the sensing element attained a steady state, the chamber was exposed to the ambient atmosphere to ensure the sensing element’s recovery behavior, and the element’s response characteristics were continuously recorded. This measurement was carried out for the desired concentrations of target analytes. The gas sensing response was determined by comparing the electrical resistance of the sensing element in dry air and in gas environment by means of Equation (2):

(2) $$S=\frac{R_{a}}{R_{v}}\;\left(\mathrm{for}\;R_{a}>>R_{v}\right)$$

where *R_a_* and *R_v_* are the resistance values of the sensing element in dry air and in presence of target gas, respectively.

## 3. Results and Discussion

### 3.1. Preparation and Characterization of Hybrids

The preparation of hybrid nanomaterials used in this study is schematically illustrated in Figure 1. Sample ZnO/TiO_2_ was prepared as follows. At first, a Zn metal plate immersed in deionized water was ablated by a millisecond pulsed laser, which is known to produce ZnO nanorods [35]. Then, the Zn plate was replaced with a Ti one to be further ablated (in presence of already formed ZnO nanorods) for another 30 min (Figure 1). In a similar way, a hybrid sample TiO_2_/ZnO was fabricated: first by ablating a Ti target in deionized water to form TiO_2_ NPs, after which a Zn target was ablated in their presence.

**Figure 2:**
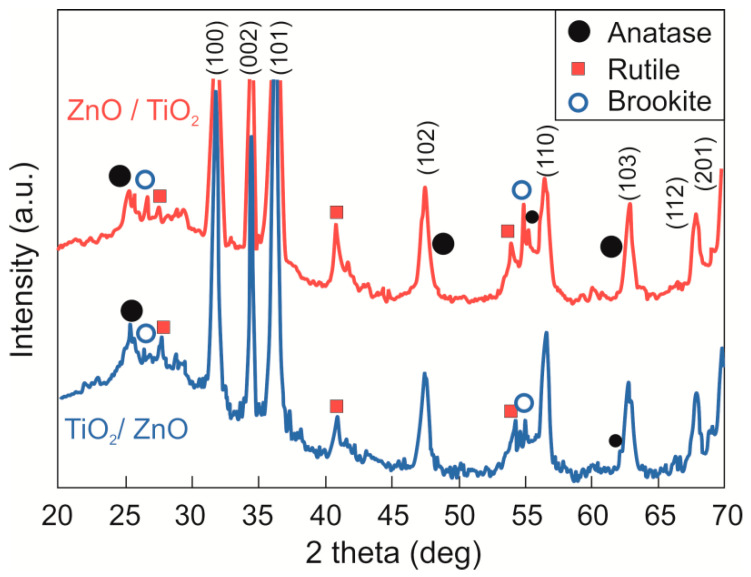
XRD patterns of samples ZnO/TiO_2_ (red) and TiO_2_/ZnO (blue).

The phase composition of as-prepared materials was studied by XRD analysis. The XRD patterns for both samples are shown in Figure 2. One can clearly see eight sharp peaks at 31.7°, 34.4°, 36.2°, 47.5°, 56.5°, 62.8°, 67.7°, and 69.8° which correspond to the planes of the hexagonal wurtzite phase of ZnO (PDF 01-089-1397). In both hybrid nanomaterials, titania is seen to show broad low-intensity peaks assigned to three crystalline phases: anatase (PDF 00-001-0562), rutile (PDF 01-072-7374), and brookite (PDF 01-076-1934), all being indicated with circles and squares in Figure 2. TiO_2_ patterns are more pronounced for sample ZnO/TiO_2_ (red pattern) where titania was produced in presence of ZnO NPs. The peaks for TiO_2_ in sample TiO_2_/ZnO are weaker, indicating lower crystallinity of titania, most probably due to secondary laser irradiation of TiO_2_ NPs during the second step of this hybrid formation.

**Figure 3:**
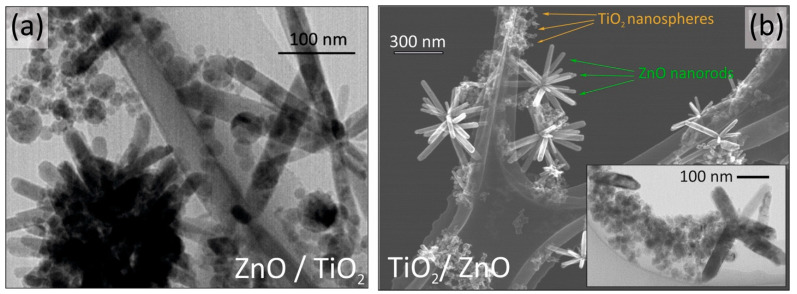
Electron microscopy images of samples (**a**) ZnO/TiO_2_ and (**b**) TiO_2_/ZnO. ZnO as nanorods and TiO_2_ as nanospheres are observed.

TEM images of samples ZnO/TiO_2_ and TiO_2_/ZnO are shown in Figure 3a,b, respectively. It is seen that in both hybrids, ZnO NPs are presented as nanorods, while TiO_2_ NPs are spherical. Both metal oxides are formed as two separate phases, which are well-mixed in homogeneous solid materials. Previously, Honda et al. reported on size-controlled ZnO nanorods prepared under different conditions by means of a millisecond laser (pulse width of 0.5, 1, and 2 ms and peak energy of 1 and 5 kW) [35]. According to Figure 3b, the formation and growth of ZnO NPs in titania-containing medium (sample TiO_2_/ZnO) tends to provide ZnO rods with a larger aspect ratio (longer and thinner nanorods), more specifically, with their length varying from 50 to 190 nm and their width ranging from 10 to 40 nm. Meanwhile, bigger and uniform ZnO nanorods with a length of 120–210 nm and width 25–55 nm were formed in sample ZnO/TiO_2_ where ZnO NPs were then irradiated during the second stage for another 30 min. This can be explained by a longer heating period of the ZnO nanorods formed in sample ZnO/TiO_2_, in good agreement with previous reports [15,35].

**Figure 4:**
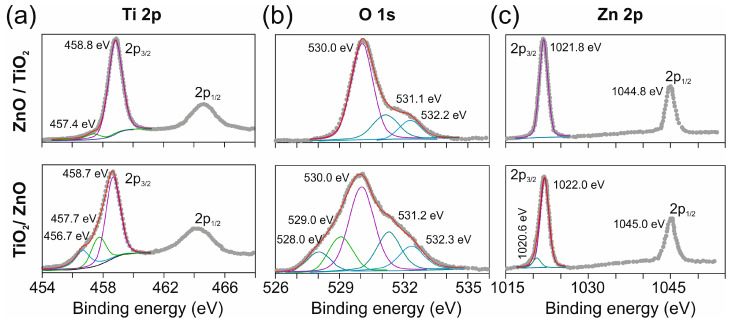
Ti 2p (**a**), O 1s (**b**), and Zn 2p (**c**) XPS spectra of hybrid materials: ZnO/TiO_2_ (top row) and TiO_2_/ZnO (bottom row). Pink, green, and light blue fitting peaks in (**a**) are related to Ti^4+^, Ti^3+^, and Ti^2+^ species, respectively. Pink, grey, light blue, light green, and dark blue fitting peaks in (**b**) are related to oxide ions from O^2−^-Ti^4+^ and O^2−^-Zn^2+^ bonds, oxygen vacancies, surface hydroxyl groups, O^2−^-Ti^3+^, and O^2−^-Ti^2+^ bonds, respectively.

Ablation of metallic Ti in water was typically reported to produce spherical TiO_2_ NPs [22,23,26]. The diameter of such TiO_2_ NPs observed in sample ZnO/TiO_2_ is in a narrower range (5–30 nm) than those formed in sample TiO_2_/ZnO (5–65 nm) [15]. This can probably be attributed to the presence of ZnO nanorods in the liquid medium, which restricted the crystal growth of TiO_2_. The wider size distribution of TiO_2_ NPs in sample TiO_2_/ZnO might also be explained by secondary irradiation of TiO_2_ NPs during ablation of Zn plate in TiO_2_ dispersion (i.e., the second preparation stage) when they were subjected to additional fragmentation and melting–aggregation. Such fusion of TiO_2_ NPs is usually accompanied by a loss of crystallinity, as revealed by XRD patterns in Figure 2. A similar conclusion on the effect of secondary irradiation of titania NPs also comes from XPS data (presented in Figure 4) that confirm changes in the surface composition of analyzed NPs.

In Ti 2p XPS spectra, the Ti 2p_3/2_ and Ti 2p_1/2_ peaks are centered at 458.8 eV and 464.6 eV for sample ZnO/TiO_2_ (Figure 4a, top) and at 458.7 eV and 464.4 eV for sample TiO_2_/ZnO (Figure 4a, bottom). These values are typical for Ti^4+^ ions in titania and confirm the formation of rutile and anatase as main phases in both hybrids. As depicted in Figure 4a, the Ti 2p_3/2_ peak of material TiO_2_/ZnO is much wider than that of its counterpart ZnO/TiO_2_. It can be fitted with three Gaussian peaks: (i) a main peak at 458.7 eV assigned to Ti^4+^ ions [19,24], (ii) a peak located at lower binding energy (457.7 eV) and related to Ti^3+^ ions [20,45,46], and (iii) a lowest-energy peak at 456.7 eV associated with Ti^2+^ ions (Figure 4a, bottom) [19,23].

Based on the XPS results, we conclude that in sample TiO_2_/ZnO, its TiO_2_ NPs are more laser-modified because of longer irradiation, which leads to the formation of surface Ti^3+^ and Ti^2+^ species well seen in Figure 4a. Such species may form not only by gaining electrons from plasma, but also from Zn atoms through a redox process where surface Ti^4+^ ions get reduced. Comparison with the Ti 2p_3/2_ peak of sample ZnO/TiO_2_ (Figure 4a) shows that ablation of Ti plate in a ZnO colloid gives TiO_2_ NPs with a very low density of surface Ti^3+^ species. This conclusion is also confirmed by the O1s XPS spectra shown in Figure 4b. The O1s peak of sample ZnO/TiO_2_ was deconvoluted into three components (Figure 4b, top), with the most intensive peak at 530.0 eV assigned to O^2-^ ions in the crystal structure of both metal oxides, ZnO and TiO_2_ [47], the peak at 531.1 eV associated with surface oxygen vacancies [15], and the peak at 532.2 eV related to surface hydroxyl groups [35]. The same components, fitted at 530.0, 531.2, and 532.3 eV, are also seen in the spectrum of sample TiO_2_/ZnO (Figure 4b, bottom), where two additional peaks at lower binding energy (529.0 and 528.0 eV) appear, being associated with oxygen ions bonded to titanium in lower oxidation states, Ti(III) and Ti(II) (i.e., O^2−^-Ti^3+^ and O^2−^-Ti^2+^ bonds), in accordance with the signals for Ti^3+^ and Ti^2+^ ions observed in Ti 2p XPS spectra (Figure 4a).

The doublets consisting of Zn 2p_3/2_ and Zn 2p_1/2_ peaks are observed at 1021.8 eV and 1044.8 eV (for sample ZnO/TiO_2_) and at 1022.0 eV and 1045.0 eV (for sample TiO_2_/ZnO), (see Figure 4c). These peaks clearly indicate Zn^2+^ species in ZnO NPs available in both hybrids [35,37,47]. In addition, a weak signal is observed in sample TiO_2_/ZnO at a lower binding energy (1020.6 eV) (Figure 4c, bottom), which is most likely related to a trace amount of metallic Zn inclusions that possibly remained due to incomplete oxidation of Zn atoms in a TiO_2_-containing dispersion [35]. Thus, XPS analysis of both hybrids revealed that: (i) Zn is always presented in the form of ZnO; (ii) during the second stage of laser processing, Ti(IV) is reduced to Ti(III) and Ti(II) on the surface of sample TiO_2_/ZnO; (iii) self-doping of TiO_2_ with Ti^3+^ and Ti^2+^ is confirmed by both O1s and Ti 2p XPS spectra.

### 3.2. Gas-Sensing Properties

#### 3.2.1. Selectivity and Response Ratio

**Figure 5:**
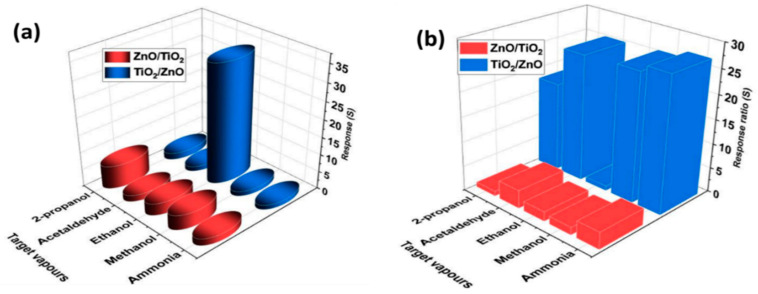
Selectivity (**a**) and selectivity factor (**b**) of samples ZnO/TiO_2_ and TiO_2_/ZnO towards 100 ppm of various analytes.

To test the gas-sensing properties of the prepared samples ZnO/TiO_2_ and TiO_2_/ZnO at room temperature, their responses towards 100 ppm of different target vapors (2-propanol, acetaldehyde, ethanol, methanol, and ammonia) were studied as shown in Figure 5a. The hybrid TiO_2_/ZnO showed a selective response towards ethanol (*S* = 34.1), whereas its ZnO/TiO_2_ counterpart exhibited selective sensing towards 2-propanol (*S* = 6.6). Response ratio [20,25], or the selectivity factor, was calculated using Equation (3), and the obtained values are presented in Figure 5b:

(3) $$Selectivity\;factor=\frac{S_{target\;gas}}{S_{Interfering\;gas}}$$

#### 3.2.2. Transient Response and Limit of Detection (LOD)

**Figure 6:**
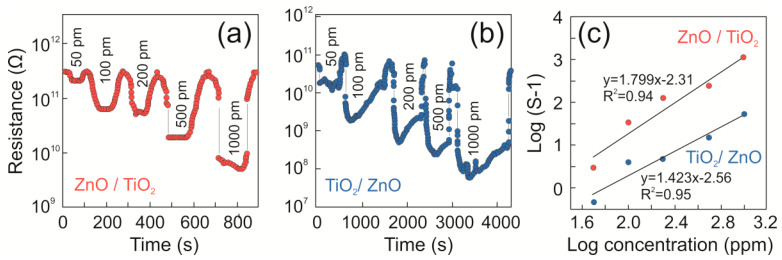
Transient resistance trends for samples (**a**) ZnO/TiO_2_ and (**b**) TiO_2_/ZnO towards varying concentrations of (**a**) 2-propanol and (**b**) ethanol. (**c**) Logarithmic response vs. logarithmic concentration trends for samples ZnO/TiO_2_ (dark blue markers) and TiO_2_/ZnO (red markers) towards 2-propanol and ethanol, respectively.

The transient resistance response of the samples in the presence of varied concentrations of 2-propanol and ethanol is shown in Figure 6a,b, respectively. The logarithmic response was observed to increase linearly with the logarithmic concentration of both 2-propanol and ethanol vapors. The observed trends were fitted to the following linear equations: *y*_(2-propanol)_ = 1.423*x* − 2.56 and *y*_(ethanol)_ = 1.799*x* − 2.31 with *R*^2^ values being 0.95 and 0.94 for 2-propanol and ethanol, respectively (see Figure 6c). Thus, the observed *R*^2^ values clearly indicate the linear relationships in both cases. Both samples ZnO/TiO_2_ and TiO_2_/ZnO showed the lowest detection limit of 50 ppm towards 2-propanol and ethanol vapors, respectively.

#### 3.2.3. Response and Recovery Times

**Figure 7:**
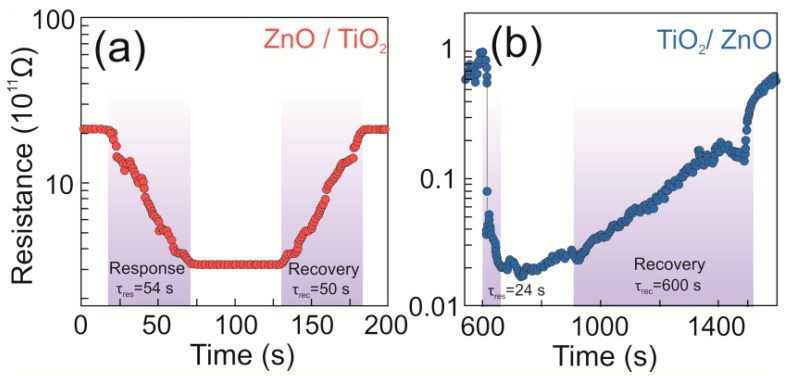
Response–recovery times of (**a**) ZnO/TiO_2_ towards 100 ppm of 2-propanol and (**b**) TiO_2_/ZnO towards 100 ppm of ethanol.

Both the response and recovery times were defined as the time taken for the sensor to reach 90% of maximum response and recover back to surface resistance in absence of target gas, respectively [48]. Such times for sample TiO_2_/ZnO were found to be 24 and 607 s, respectively, towards 100 ppm of ethanol. Meanwhile, the response and recovery times of sample ZnO/TiO_2_ were found to be 54 and 50 s, respectively, towards 100 ppm of 2-propanol (Figure 7). Though the sensor based on hybrid TiO_2_/ZnO showed a selective detection of ethanol at room temperature, its recovery time was relatively slow compared with that of its ZnO/TiO_2_ counterpart, which is probably related to the slow desorption rate of ethanol molecules at room temperature. We assume that a possible reason can be the formation of stronger hydrogen bonds between the OH group in ethanol molecules and surface hydroxyl groups available on hybrid material, while the molecule of 2-propanol experiences sterical hindrance in hydrogen bonding.

#### 3.2.4. Impact of Relative Humidity

**Figure 8:**
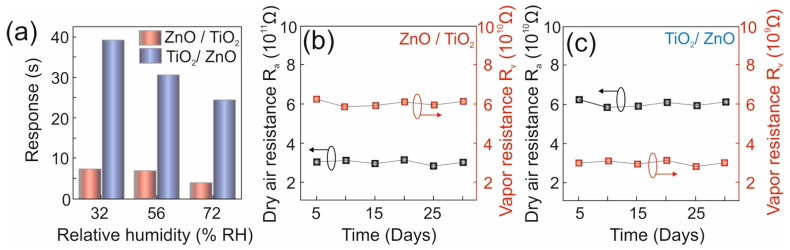
(**a**) Impact of relative humidity on sensing response. Performance stability of samples ZnO/TiO_2_ (**b**) and TiO_2_/ZnO (**c**) towards 100 ppm of 2-propanol and ethanol over time, respectively.

Relative humidity is one of the parameters known to influence sensors that operate at room temperature. Hence, the sensing response of samples ZnO/TiO_2_ and TiO_2_/ZnO towards 100 ppm of 2-propanol (or ethanol) at different% RH levels was also investigated (see Figure 8a). For this, the values of % RH were varied to 32% and 72% from the actual relative humidity of 56%, which was achieved by maintaining corresponding saturated salt solutions of MgCl_2_ and NaCl inside the sensing chamber [49]. Humidity was evaluated by a digital humidity and temperature sensor (DHT 11) integrated into the sensing chamber used [43,48]. The sensing response was found to increase by 10 and 28% at lower humidity (32% RH) for 2-propanol and ethanol, respectively (see Figure 8a). The increased sensing response observed at lower humidity is ascribed to the reduced hindrance sorption of OH^−^ ions on the surface of the sensing element, which follows the hopping charge transport behavior [50,51,52,53]. At the same time, the response was decreased by 44 and 20% as shown in Figure 8a at higher humidity (of 72%RH) for 2-propanol and ethanol, respectively. This may be attributed to the reduced sorption process in the presence of excess OH^−^ ions on the surface of the sensing element, which follows the Grotthuss physisorption charge transfer mechanism [50,51,52,53].

#### 3.2.5. Stability of Performance over Time

The stability of sensors based on samples ZnO/TiO_2_ and TiO_2_/ZnO was evaluated as their sensing was tested towards 100 ppm of corresponding target gases over a period of 30 days, during which the sensors were tested every 5 days. Both sensing elements are seen in Figure 8b,c to show good reproducible results with minimal changes in *R_a_* and *R_v_* after 30 days. This reveals the long-term stability of both hybrids ZnO/TiO_2_ and TiO_2_/ZnO as they were subjected to multiple sorption–desorption cycles.

#### 3.2.6. Sensing Mechanism

The band gap of TiO_2_ (3.2 eV) is smaller than that of ZnO (3.3 eV) and the electron affinity of TiO_2_ (4.2 eV) is larger than that of ZnO (4.1 eV), which predicts the formation of *n-n* homo-junction at their interface. Isolated bands of materials ZnO and TiO_2_ should exist in each material and a heterojunction barrier is generated at their interface during contact. The surface resistance related to the heterojunction barrier can be expressed by Equation (4):

(4) $$R\propto B\;\exp\left(\frac{q\Phi }{kT}\right)$$

where *R* is the resistance corresponding to heterojunction barrier, *B* is the constant related to ambient temperature, Φ is the heterojunction barrier, *T* is the absolute temperature and *k* is the Boltzmann constant.

When a sensing element is maintained in the ambient atmosphere, it experiences chemisorption of atmospheric oxygen on its surface (Equations (5) and (6)). The adsorption of conduction band electrons of the sensing element by atmospheric oxygen molecules results in the increased space charge width around each grain. This process results in increased surface resistance and the latter resistance is considered the baseline for sensing measurements. Upon interaction with reducing type target analytes, the addition of conduction band electrons to the sensing element results in the reduction of space charge width. In turn, this reduces the surface resistance of the sensing element (*R_a_*). The possible vapor solid interactions involved are expressed in Equations (7) and (8). During the desorption process, the surface resistance of the sensing element again reaches its baseline as a reversible process.

(5) $$O_{2}\left(atm\;air\right)⇋\;O_{2\;}\left(ads\right)$$

(6) $$O_{2\;}\left(ads\;\right)+e^{-}⇋\;O_{2}{}^{-}\left(ads\right)$$

(7) $$C_{2}H_{5}OH+30_{2\;\left(\mathrm{Ti}O_{2}/\mathrm{ZnO}\;surface\right)}^{-}⇌\;2CO_{2}↑+3H_{2}O↑+3e^{-}↓$$

(8) $$2C_{3}H_{8}O+90_{2\;\left(\mathrm{ZnO}/\mathrm{Ti}O_{2}surface\right)}^{-}⇌\;6CO_{2}↑+8H_{2}O↑+9e^{-}↓$$

Table 1 compares the sensing performance of hybrid materials prepared in the present work with that of similar materials previously reported in the literature. It is seen that, in comparison with their counterparts previously reported in the literature, samples ZnO/TiO_2_ and TiO_2_/ZnO are seen to exhibit selective detection of propanol and ethanol at room temperature.

## 4. Conclusions

In this work, using the green preparation method of pulsed laser ablation in the liquid phase, we prepared two hybrid nanomaterials based on ZnO and TiO_2_. To produce such samples, Zn and Ti metal plates were ablated in water in different sequences: (i) Zn target followed by Ti target in presence of already formed ZnO nanoparticles and (ii) Ti target followed by Zn target in presence of already formed TiO_2_ nanoparticles. Both hybrid materials were found to have spherical TiO_2_ and rod-like ZnO nanoparticles as their components, demonstrating similar morphology irrespective of the preparation approaches. However, the surface chemistry of both materials was affected by laser irradiation time and liquid media, showing the formation of reduced oxidation states (+3) and (+2) of Ti in parallel with Ti(+4) in TiO_2_. When used as chemiresistor gas sensing elements at room temperature, these hybrids showed selectivity either to ethanol or 2-propanol and long-term stability performance. Thus, laser-prepared hybrid metal oxide nanomaterials are shown to demonstrate selective gas sensing depending on their preparation protocol.

## Figures and Tables

**Table 1:** Comparison with other TiO_2_-ZnO sensors previously reported in the literature.

Materials	Operating Temperature (°C)	Target Gas	Response (S)	Detection Limit (ppm)	Response/Recovery Time (s)	Reference
TiO_2_/ZnO heterostructure nanowire	200	NO_2_	7	1-40	65/98	[54]
ZnO nanowires on TiO_2_/ZnO nanocomposite	230	Ethanol	16.82	200	4/6	[55]
ZnO/TiO_2_–PANI	Room temperature	LPG NO_2_	87 412	2000 20	99/118 87/79	[56]
TiO_2_ nanotube arrays by ZnO	300	H_2_	340	100	22/-	[57]
TiO_2_-modified ZnO tetrapods	350	CO	106	50	-/-	[58]
Zn_2_TiO_4_	Room temperature	Propanol	~17	500–3000	-/-	[59]
TiO_2_/ZnO hybrid	Room temperature	Ethanol	34.1	50	24/607	Present work
ZnO/TiO_2_ hybrid	Room temperature	2-propanol	6.6	50	54/50	Present work

## Data Availability

The data presented in this study are available on request from the corresponding authors.

## References

[1] Kim M.J., Osone S., Kim T.S., Higashi H., Seto T. (2017). Synthesis of nanoparticles by laser ablation: A review. KONA Powder Part. J..

[2] Farkhutdinova E.D., Shabalina A.V., Gerasimova M.A., Nemoykina A.L., Vodyankina O.V., Svetlichnyi V.A. (2020). Highly defective dark nano titanium dioxide: Preparation via pulsed laser ablation and application. Materials.

[3] Shabalina A.V., Golubovskaya A.G., Fakhrutdinova E.D., Kulinich S.A., Vodyankina O.V., Svetlichnyi V.A. (2022). Phase and Structural Thermal Evolution of Bi–Si–O Catalysts Obtained via Laser Ablation. Nanomaterials.

[4] Zeng H.B., Du X.W., Singh S.C., Kulinich S.A., Yang S.K., He J.P., Cai W.P. (2012). Nanomaterials via laser ablation/irradiation in liquid: A review. Adv. Funct. Mater..

[5] Gurbatov S.O., Puzikov V., Storozhenko D., Modin E., Mitsai E., Cherepakhin A., Shevlyagin A., Gerasimenko A.V., Kulinich S.A., Kuchmizhak A.A. (2023). Multigram-scale production of hybrid Au-Si nanomaterial by laser ablation in liquid (LAL) for temperature-feedback optical nano-sensing, light-to-heat conversion and anti-counterfeit labeling. ACS Appl. Mater. Interfaces.

[6] Zhang D., Liu J., Li P., Tian Z., Liang C. (2017). Recent advances in surfactant-free, surface-charged, and defect-rich catalysts developed by laser ablation and processing in liquids. ChemNanoMat.

[7] Shabalina A.V., Svetlichnyi V.A., Kulinich S.A. (2022). Green laser ablation-based synthesis of functional nanomaterials for generation, storage and detection of hydrogen. Curr. Opin. Green Sustain. Chem..

[8] Mintcheva N., Srinivasan P., Rayappan J.P.P., Kuchmizhak A.A., Gurbatov S., Kulinich S.A. (2020). Room-temperature gas sensing of laser-modified anatase TiO_2_ decorated with Au nanoparticles. Appl. Surf. Sci..

[9] Shabalina A.V., Fakhrutdinova E.D., Golubovskaya A.G., Kuzmin S.M., Koscheev S.V., Kulinich S.A., Svetlichnyi V.A., Vodyankina O.V. (2022). Laser-assisted preparation of highly-efficient photocatalytic nanomaterial based on bismuth silicate. Appl. Surf. Sci..

[10] Gavrilenko E.A., Goncharova D.A., Lapin I.N., Nemoykina A.L., Svetlichnyi V.A., Aljulaih A.A., Mintcheva N., Kulinich S.A. (2019). Comparative study of physicochemical and antibacterial properties of ZnO nanoparticles prepared by laser ablation of Zn target in water and air. Materials.

[11] Mintcheva N., Aljulaih A.A., Bito S., Honda M., Kondo T., Iwamori S., Kulinich S.A. (2018). Nanomaterials produced by laser beam ablating Sn-Zn alloy in water. J. Alloys Compd..

[12] Nemoykina A.L., Shabalina A.V., Svetlichnyi V.A. (2019). Restoration and conservation of old low-quality book paper using aqueous colloids of magnesium oxyhydroxide obtained by pulsed laser ablation. J. Cult. Herit..

[13] Niu K.Y., Kulinich S.A., Yang J., Zhu A.L., Du X.W. (2012). Galvanic replacement reactions of active metal nanoparticles. Chem.–Eur. J..

[14] Shankar P., Ishak M.Q.H., Padarti J.K., Mintcheva N., Iwamori S., Gurbatov S.O., Lee J.H., Kulinich S.A. (2020). ZnO@graphene oxide core@shell nanoparticles prepared via one-pot approach based on laser ablation in water. Appl. Surf. Sci..

[15] Mintcheva N., Yamaguchi S., Kulinich S.A. (2020). Hybrid TiO_2_-ZnO nanomaterials prepared by laser ablation in liquid method. Materials.

[16] Tarasenka N., Shustava E., Butsen A., Kuchmizhak A.A., Pashayan S., Kulinich S.A., Tarasenko N. (2021). Laser-assisted fabrication and modification of copper and zinc oxide nanostructures in liquids for photovoltaic applications. Appl. Surf. Sci..

[17] Nikolov A.S., Atanasov P.A., Milev D.R., Stoyanchov T.R., Deleva A.D., Peshev Z.Y. (2009). Synthesis and characterization of TiO_x_ nanoparticles prepared by pulsed-laser ablation of Ti target in water. Appl. Surf. Sci..

[18] Amin M., Tomko J., Naddeo J.J., Jimenez R., Bubb D.M., Steiner M., Fitz-Gerald J., O’Malley S.M. (2015). Laser-assisted synthesis of ultra-small anatase TiO_2_ nanoparticles. Appl. Surf. Sci..

[19] Huang C.N., Bow J.S., Zheng Y., Chen S.Y., Ho N., Shen P. (2010). Nonstoichiometric titanium oxides via pulsed laser ablation in water. Nanoscale Res. Lett..

[20] García Guillén G., Shaji S., Mendivil I., Avellaneda D., Castillo G., Roy T., Garcia-Gutierrez D., Krishnan B. (2017). Effects of ablation energy and post-irradiation on the structure and properties of titanium dioxide nanomaterials. Appl. Surf. Sci..

[21] Singh S.C., Swarnkar R.K., Gopal R. (2009). Synthesis of titanium dioxide nanomaterial by pulsed laser ablation in water. J. Nanosci. Nanotechnol..

[22] Hong S.M., Lee S., Jung H.J., Yu Y., Shin J.H., Kwon K.Y., Choi M.Y. (2013). Simple preparation of anatase TiO_2_ nanoparticles via pulsed laser ablation in liquid. Bull. Korean Chem. Soc..

[23] Barreca F., Acacia N., Barletta E., Spadaro D., Curro G., Neri F. (2010). Small size TiO_2_ nanoparticles prepared by laser ablation in water. Appl. Surf. Sci..

[24] Chaturvedi A., Joshi M.P., Mondal P., Sinha A.K., Srivastava A.K. (2017). Growth of anatase and rutile phase TiO_2_ nanoparticles using pulsed laser ablation in liquid: Influence of surfactant addition and ablation time variation. Appl. Surf. Sci..

[25] Liu P., Cai W., Fang M., Li Z., Zeng H., Hu J., Luo X., Jing W. (2009). Room temperature synthesized rutile TiO_2_ nanoparticles induced by laser ablation in liquid and their photocatalytic activity. Nanotechnology.

[26] Boutinguiza M., Rodríguez-González B., del Val J., Comesaña R., Lusquiños F., Pou J. (2011). Laser-assisted production of spherical TiO_2_ nanoparticles in water. Nanotechnology.

[27] Boutinguiza M., Rodríguez-González B., del Val J., Comesaña R., Lusquiños F., Pou J. (2012). Production of TiO_2_ crystalline nanoparticles by laser ablation in ethanol. Appl. Surf. Sci..

[28] Cho J.M., Song J.K., Park S.M. (2009). Characterization of ZnO nanoparticles grown by laser ablation of a Zn target in neat water. Bull. Korean Chem. Soc..

[29] Dorranian D., Solati E., Dejam L. (2012). Photoluminescence of ZnO nanoparticles generated by laser ablation in deionized water. Appl. Phys. A.

[30] Goto T., Honda M., Kulinich S.A., Shimizu Y., Ito T. (2015). Defects in ZnO nanoparticles laser-ablated in water-ethanol mixture at different pressures. Jpn. J. Appl. Phys..

[31] Kondo T., Sato Y., Kinoshita M., Shankar P., Mintcheva N.N., Honda M., Iwamori S., Kulinich S.A. (2017). Room temperature ethanol sensor based on ZnO prepared via laser ablation in water. Jpn. J. Appl. Phys..

[32] Zamiri R., Zakaria A., Ahangar H.A., Darroudi M., Zak A.K., Drummen G.P.C. (2012). Aqueous starch as a stabilizer in zinc oxide nanoparticle synthesis via laser ablation. J. Alloys Compd..

[33] Kawabata K., Nanai Y., Kimura S., Okuno T. (2012). Fabrication of ZnO nanoparticles by laser ablation of sintered ZnO in aqueous solution. Appl. Phys. A.

[34] Usui H., Shimizu Y., Sasaki T., Koshizaki N. (2005). Photoluminescence of ZnO nanoparticles prepared by laser ablation in different surfactant solutions. J. Phys. Chem. B.

[35] Honda M., Goto T., Owashi T., Rozhin A.G., Yamaguchi S., Ito T., Kulinich S.A. (2016). ZnO nanorods prepared via ablation of Zn with millisecond laser in liquid media. Phys. Chem. Chem. Phys..

[36] Abbas K.N., Bidin N. (2017). Morphological driven photocatalytic activity of ZnO nanostructures. Appl. Surf. Sci..

[37] Mintcheva N., Aljulaih A.A., Wunderlich W., Kulinich S.A., Iwamori S. (2018). Laser-ablated ZnO nanoparticles and their photocatalytic activity towards organic pollutants. Materials.

[38] Kubiak A., Siwinska-Ciesielczyk K., Jesionowski T. (2018). Titania-based hybrid materials with ZnO, ZrO_2_ and MoS_2_: A review. Materials.

[39] Zhang X., Yuan J., Zhu J., Fan L., Chen H., He H., Wang Q. (2019). Visible light photocatalytic performance of laser-modified TiO_2_/SnO_2_ powders decorated with SiC nanocrystals. Ceram. Int..

[40] Gondal M.A., Ilyas A.M., Fasasi T.A., Dastageer M.A., Seddigi Z.S., Qahtan T.F., Faiz M., Khattak G.D. (2015). Synthesis of green TiO_2_/ZnO/CdS hybrid nano-catalyst for efficient light harvesting using an elegant pulsed laser ablation in liquids method. Appl. Surf. Sci..

[41] Lee B.H., Nakayama T., Tokoi Y., Suzuki T., Niihara K. (2011). Synthesis of CeO_2_/TiO_2_ nanoparticles by laser ablation of Ti target in cerium (III) nitrate hexahydrate (Ce(NO_3_)_3_·6H_2_O) aqueous solution. J. Alloys Compd..

[42] Gondal M.A., Ilyas A.M., Baig U. (2016). Pulsed laser ablation in liquid synthesis of ZnO/TiO_2_ nanocomposite catalyst with enhanced photovoltaic and photocatalytic performance. Ceram. Int..

[43] Shankar P., Rayappan J.B.B. (2016). Racetrack, Effect on the dissimilar sensing response of ZnO thin film—An anisotropy of isotropy. ACS Appl. Mater. Interfaces.

[44] Srinivasan P., Kulandaisamy A.J., Mani G.K., Babu K.J., Tsuchiya K., Rayappan J.B.B. (2019). Development of an acetone sensor using nanostructured Co_3_O_4_ thin films for exhaled breath analysis. RSC Adv..

[45] Pan S.S., Lu W., Zhao W.H., Tong W., Li M., Jin L.M., Choi J.Y., Qi F., Chen S.G., Fei L.F. (2013). Self-doped rutile titania with high performance for direct and ultrafast assay of H_2_O_2_. ACS Appl. Mater. Interfaces.

[46] Zhou S., Liu S., Su K., Jia K. (2019). Facile synthesis of Ti^3+^ self-doped and sulfur-doped TiO_2_ nanotube arrays with enhanced visible-light photoelectrochemical performance. J. Alloy. Compd..

[47] Yang L., Zhao Q., Willander M., Liu X., Fahlman M., Yang J.H. (2010). Origin of the surface recombination centers in ZnO nanorods arrays by X-ray photoelectron spectroscopy. Appl. Sur. Sci..

[48] Srinivasan P., Rayappan J.B.B. (2019). Highly crystalline {010} facet grown α-MoO_3_ nanobelts for resistive sensing of n-butanol vapor at room temperature. Microchim. Acta.

[49] Sharma N., Sharma N., Srinivasan P., Kumar S., Rayappan J.B.B., Kailasam K. (2018). Heptazine based organic framework as a chemiresistive sensor for ammonia detection at room temperature. J. Mater. Chem. A.

[50] Mani G.K., Rayappan J.B.B. (2014). Novel and facile synthesis of randomly interconnected ZnO nanoplatelets using spray pyrolysis and their room temperature sensing characteristics. Sens. Actuators B.

[51] Barsan N., Schweizer-Berberich M., Göpel W. (1999). Fundamental and practical aspects in the design of nanoscaled SnO_2_ gas sensors: A status report. J. Anal. Chem..

[52] Barsan N., Weimar U. (2001). Conduction model of metal oxide gas sensors. J. Electroceram..

[53] Sears W.M. (2000). Effect of oxygen stoichiometry on the humidity sensing characteristics of bismuth iron molybdate. Sens. Actuators B.

[54] Ramgir N., Bhusari R., Rawat N.S., Patil S.J., Debnath A.K., Gadkari S.C., Muthe K.P. (2020). TiO_2_/ZnO heterostructure nanowire based NO_2_ sensor. Mater. Sci. Semicond. Process..

[55] Hashemi M.M., Nikfarjam A., Hajghassem H., Salehifar N. (2020). Hierarchical dense array of ZnO nanowires spatially grown on ZnO/TiO_2_ nanofibers and their ultraviolet activated gas sensing properties. J. Phys. Chem. C.

[56] Sonker R.K., Yadav B.C., Gupta V., Tomar M. (2019). Fabrication and characterization of ZnO-TiO_2_-PANI (ZTP) micro/nanoballs for the detection of flammable and toxic gases. J. Hazard. Mater..

[57] Yu A., Xun H., Yi J. (2019). Improving hydrogen sensing performance of TiO_2_ nanotube arrays by ZnO modification. Front. Mater..

[58] Santhaveesuk T., Shimanoe K., Suematsu K., Choopun S. (2018). Size-independent and ultrahigh CO gas sensor based on TiO_2_ modified ZnO tetrapods. Phys. Status Solidi.

[59] Gaidan I., Brabazon D., Ahad I.U. (2017). Response of a Zn_2_TiO_4_ gas sensor to propanol at room temperature. Sensors.

